# Can COVID-19 impact the natural history of paracoccidioidomycosis? Insights from an atypical chronic form of the mycosis

**DOI:** 10.1590/S1678-9946202365057

**Published:** 2023-12-01

**Authors:** César Augusto Tomaz de Souza, Cesar Cilento Ponce, Gisele Burlamaqui Klautau, André Nathan Costa, Wladimir Queiroz, Rosely Antunes Patzina, Gil Benard, José Angelo Lauletta Lindoso

**Affiliations:** 1 Instituto de Infectologia Emilio Ribas, São Paulo, São Paulo, Brazil; 2 Instituto Adolfo Lutz, São Paulo, São Paulo, Brazil; 3 Santa Casa de São Paulo, Faculdade de Medicina, São Paulo, São Paulo, Brazil; 4 Universidade de São Paulo, Faculdade de Medicina, Departamento de Cardio-Pneumologia, São Paulo, São Paulo, Brazil; 5 Universidade de São Paulo, Faculdade de Medicina, Instituto de Medicina Tropical de São Paulo, Laboratório de Micologia Médica (LIM-53), São Paulo, São Paulo, Brazil; 6 Universidade de São Paulo, Faculdade de Medicina, Instituto de Medicina Tropical de São Paulo, Laboratório de Protozoologia (LIM-49), São Paulo, São Paulo, Brazil

**Keywords:** Paracoccidioidomycosis, COVID-19, Atypical clinical form, Esophagus

## Abstract

Paracoccidioidomycosis (PCM) is a systemic fungal infection caused by *Paracoccidioides* spp. It can occur as an acute/subacute form (A/SAF), a chronic form (CF) and rarely as a mixed form combining the features of the two aforementioned forms in an immunocompromised patient. Here, we report a 56-year-old male patient with CF-PCM who presented with atypical manifestations, including the development of an initial esophageal ulcer, followed by central nervous system (CNS) lesions and cervical and abdominal lymphatic involvement concomitant with severe SARS-CoV-2 infection. He was HIV-negative and had no other signs of previous immunodeficiency. Biopsy of the ulcer confirmed its mycotic etiology. He was hospitalized for treatment of COVID-19 and required supplemental oxygen in the intensive unit. The patient recovered without the need for invasive ventilatory support. Investigation of the extent of disease during hospitalization revealed severe lymphatic involvement typical of A/SAF, although the patient`s long history of high-risk exposure to PCM, and lung involvement typical of the CF. Esophageal involvement is rare in non-immunosuppressed PCM patients. CNS involvement is also rare. We suggest that the immunological imbalance caused by the severe COVID-19 infection may have contributed to the patient developing atypical severe CF, which resembles the PCM mixed form of immunosuppressed patients. Severe COVID-19 infection is known to impair the cell-mediated immune response, including the antiviral response, through T-lymphopenia, decreased NK cell counts and T-cell exhaustion. We hypothesize that these alterations would also impair antifungal defenses. Our case highlights the potential influence of COVID-19 on the course of PCM. Fortunately, the patient was timely treated for both diseases, evolving favorably.

## INTRODUCTION

First described in 1908 by Adolpho Lutz, paracoccidioidomycosis (PCM) is a systemic granulomatous disease caused by fungi of the *Paracoccidoides brasiliensis* complex (*P. brasiliensis and P. lutzii*)^1^. The disease comprises two well defined clinical presentations: an acute/subacute form (A/SAF), which develops soon after exposure to *Paracoccidioides* spp., preferentially in adult males^2^, and a chronic form (CF), which develops many years or decades after exposure to the fungi, due to the reactivation of subclinical foci, and occurs more frequently in adult males^2^. The factors that drive these different outcomes are unknown, but likely involve the host’s immune response. Both in its A/SA and C forms, this mycosis can affect multiple organs: the A/SAF mainly affects those associated with the reticuloendothelial system (lymph node chains, spleen, liver and bone marrow) and the skin, and the CF mostly impacts the lungs, larynx, upper respiratory tract, adrenals, skin, and central nervous system^1,2^.

The disease caused by SARS-CoV-2, the agent of COVID-19, ranges from a mild or asymptomatic respiratory infection to severe respiratory distress^3^. After reaching tropical areas, COVID-19 was expected to overlap with local endemic infectious diseases. However, the literature only reports seven patients with ongoing PCM who developed COVID-19, and all cases from a single health center^4,5^. Interestingly, six of these patients had the A/SAF and only one had the CF. This situation may simply reflect the epidemiological characteristics of the patients treated in this center, rather than an immune alteration induced by SARS-CoV-2 that would promote the development of the A/SAF or exacerbation of the PCM. It is likely that a significant proportion of Brazilian patients with PCM were exposed to or developed COVID-19^4,5^. However, this co-infection was rarely mentioned in the literature, probably because the two infections did not influence each other’s courses. PCM and COVID-19 can share clinical signs related to pulmonary involvement, and both are influenced by patients’ immune responses. SARS-CoV-2 infection was shown to increase the risk of invasive fungal infections, probably by altering the host’s cellular immune response. The association with invasive aspergillosis or severe mucormycosis has frequently been described in patients with COVID-19^6^. In this article, we reported the rare case of a patient with a CF of PCM that involved the esophagus, and whose mycotic disease evolved atypically with CNS lesions and some manifestations that mimic those of the A/SAF. We hypothesize that the atypical course of PCM in this patient could be ascribed, at least in part, to the immunological imbalance caused by the severe SARS-CoV-2 co-infection.

## CASE REPORT

This study was approved by the Research Ethics Committee of the Emilio Ribas Infectology Institute (report Nº 5.310.289).

**Figure 1:**
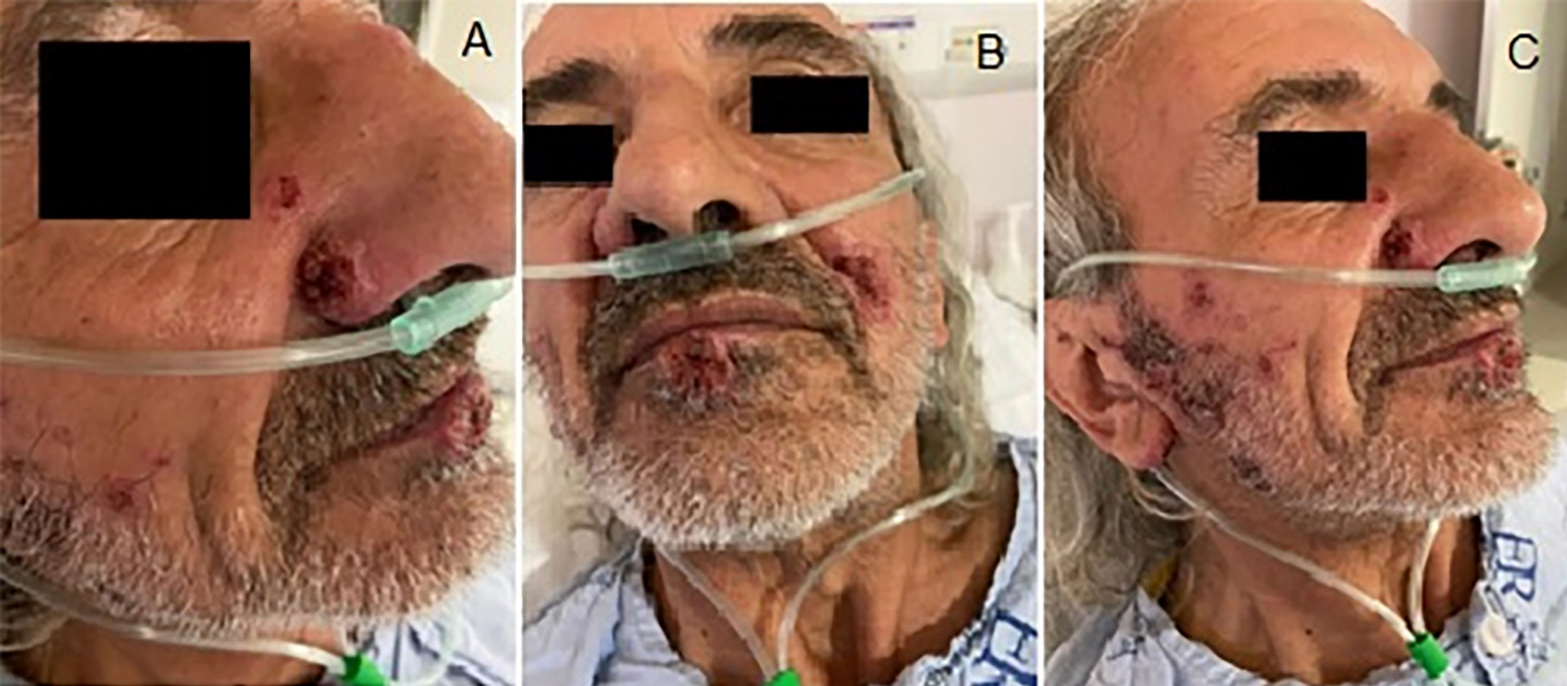
- Ulcerated and ulcer-crusted facial lesions in the temporomandibular area, lower lip, ear lobe, right nasal wing and jaw.

**Figure 2:**
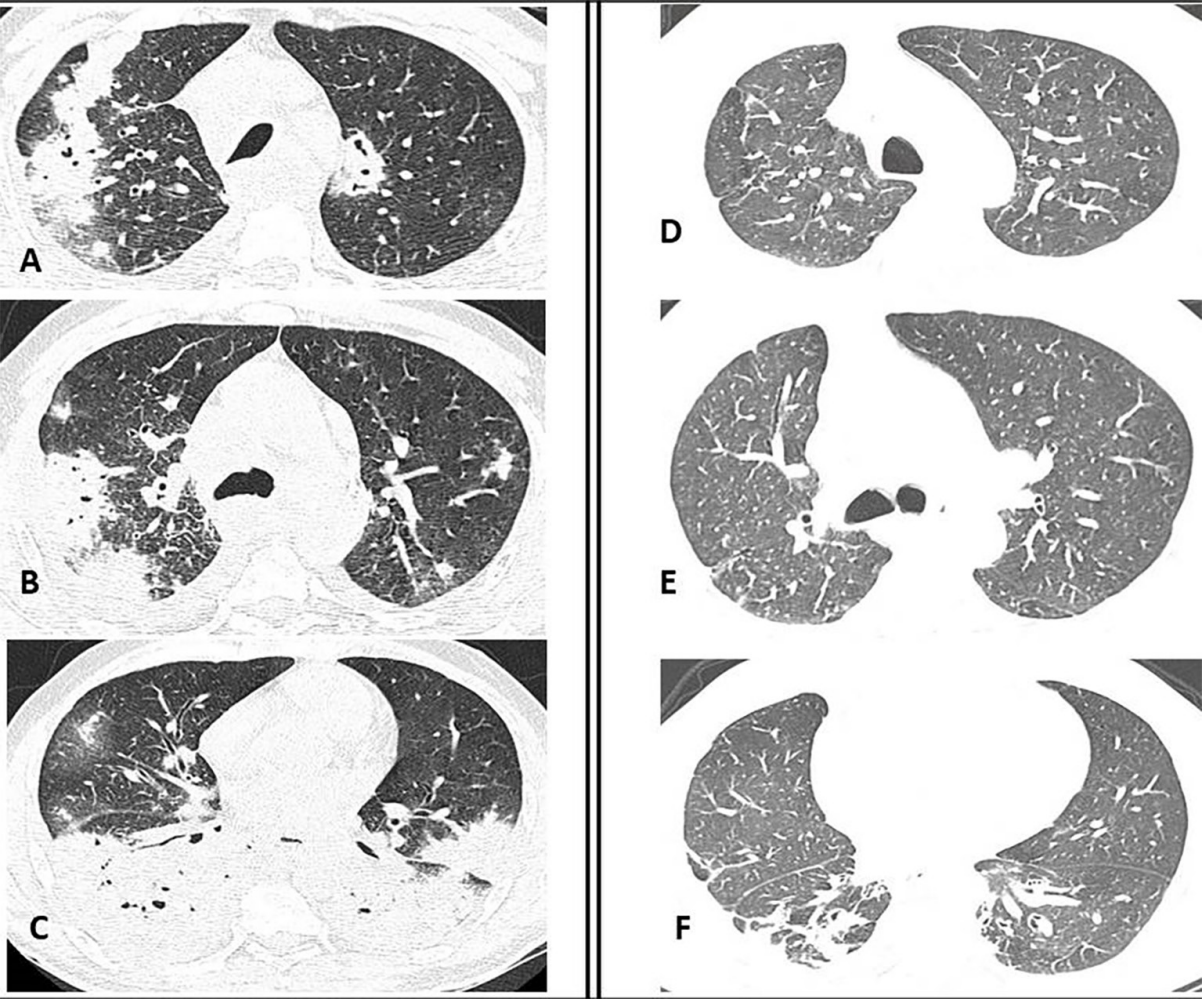
- Mixed from PCM in a 56-year-old man. High-resolution computed tomography scan (CT) before (A,B,C) and after treatment (D,E,F); A) CT at the level of the upper lobes, depicting patchy ground-glass attenuation, ill-defined opacities, airspace consolidation and cavitary lesions; B) CT at the level of the carina, showing irregular airspace consolidations with associated cavitations, nodules surrounded by ground glass, and interlobular septal thickening; C) CT at the lower lobe level, showing bilateral consolidations with associated multiple confluent nodules; D) CT at the level of the upper lobes, showing mosaic attenuation and septal thickening; E) CT at the level of the carina, depicting septal thickening and peripheral ill-defined opacities; F) CT al the lower lobe level, showing architectural distortion, residual peripheral consolidations and traction bronchiectasis.

**Figure 3:**
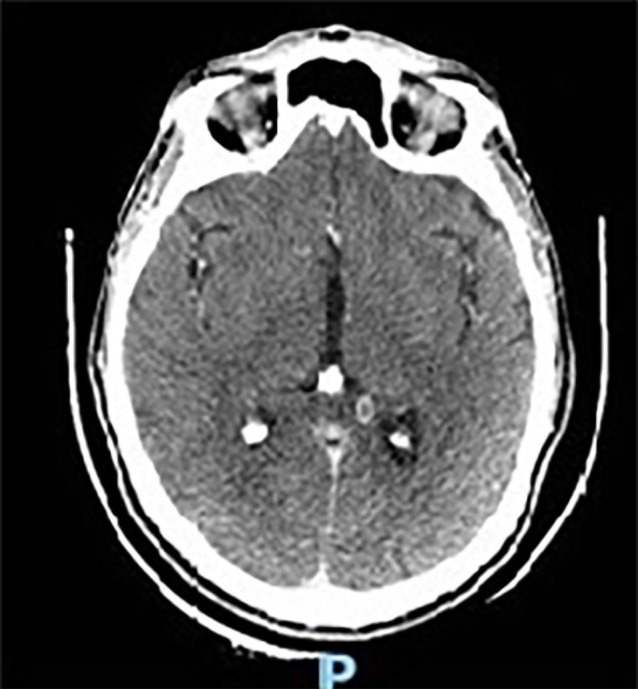
- High-resolution computed tomography scan of the brain showing a ring-enhancing lesion in the splenium of the left corpus callosum.

A 56-year-old white male who had been living in a rural area (Mairipora city) of the Sao Paulo State for over 30 years, searched for at a health care center complaining about 3-month progressive dyspepsia and dysphagia, which were followed by a 17 kg weight loss and the development of multiple cutaneous lesions in the cephalic segment. He also reported a history of dry cough that had persisted for at least a year, which he attributed to his cigarette smoking habit (15 packs per year). He underwent a digestive endoscopy, which revealed no specific alterations in his stomach and duodenum but did show an esophageal ulcerated lesion with imprecise limits, friable to the touch. The ulcer was then biopsied. Subsequently, the patient’s dyspnea worsened, he tested positive for SARS-CoV-2, and was referred to our service. On admission, he presented the following vital signs: 84% oxygen saturation (SatO2) in room air, 100/60 mmHg blood pressure, 82 beats/min heart rate, and 20 breaths/min respiratory rate. He had cutaneous lesions in the temporomandibular area, lower lip, ear lobe, right nasal wing and jaw, compatible with those commonly caused by PCM (Figure 1). No lymph node enlargements were palpable on admission. A laboratory screening found the following results: hemoglobin, 12.1 g/dL; leukocytes, 12,300/mm3; platelets, 447,000 mm^3^; C-reactive protein: 288 mg/dL and a negative HIV serology. The sputum culture and acid-fast bacilli smear yielded negative results, as well as the PCR for *Mycobacterium tuberculosis*. A computed tomographic (CT) scan of the patient’s chest revealed extensive consolidations in the upper right lobe and in both lower lobes, some of which were excavated, along with areas of ground glass opacities, diffuse septal thickening and small right pleural effusion (Figures 2A, 2B and 3C). The abdominal CT scan revealed multiple necrotic mesenteric and gastro-hepatic lymph nodes, which measured up to 3.7 cm, and nodular thickening of both adrenal glands. The cranial CT scan found multiple ring-enhancing lesions, which were more pronounced in the splenium of the left corpus callosum (Figure 3). During hospitalization, the patient was transferred to the ICU to receive supplemental O2 and piperacillin/tazobactam (total, 7 days) to treat the respiratory COVID-19 syndrome. No invasive ventilatory support was required. His respiratory condition improved and after 7 days he returned to the ward to continue treatment. At that time, lymph node enlargements in the left submandibular region had become palpable. A lymph node biopsy revealed a granulomatous inflammation and the Grocott stain technique revealed structures compatible with *Paracoccidioides* sp*.* infection. At that same time, we received the report of the prior esophageal ulcer biopsy, which also showed a loose, granulomatous-like inflammation with giant cells and yeast-like cells, suggestive of *Paracoccidoides* sp. A serology test for PCM was then performed and yielded a highly positive result (1:128). Treatment with liposomal amphotericin B was started (5 mg/kg/day), which resulted in marked clinical improvement after 3 weeks. He was discharged after 41 days of hospitalization receiving sulfamethoxazole/trimethoprim (800 mg/160 mg every 8h), with resolution of the skin and mucosal lesions, and improvement of the respiratory symptoms. During the outpatient follow-up, a cranial nuclear magnetic resonance revealed complete resolution of the CNS parenchymal lesions, and a chest CT scan showed marked improvement of the pulmonary parenchyma alterations, with only residual lesions left, mainly in the right lower lobe (Figures 2D, 2E and 2F). Marked improvements in the superficial and gastrointestinal lymphatic involvement were also noted.

## DISCUSSION

We presented the case of a patient who developed an atypical chronic form of PCM and was hospitalized due to a severe SARS-CoV-2 co-infection. Several points regarding the clinical presentation and possible pathogenesis of this case deserve to be mentioned. First, esophageal involvement rarely occurs in non-immunocompromised patients with PCM—we were able to retrieve only seven cases of this occurrence in the literature, all except one in patients with the CF of PCM^7-12^. The typical pattern in the CF of PCM is the development of oropharyngeal lesions, which represents the farthest localization reached by the fungal spread through the lymphatic route^13^. In these cases, the fungi arise from the primary pulmonary foci and spread through the intrapulmonary lymph nodes, hilar nodes, and subsequently the paratracheal nodes, reaching the mucosa through inverse lymphatic flow. Radiology imaging evidences compromised deep lymphatic chains underlying the mucosa and other localized lesions^14-16^. For unknown reasons, in the patient described here, the lymphatic spread alternatively drove the fungi to the esophagus, sparing the oropharyngeal mucosa. In the single report of an A/SAF patient with esophageal lesions in the literature, this involvement was, differently from the esophageal lesions of CF patients, probably due to contiguous spread, since the lesions were described as secondary to mediastinal lymph node enlargement that fistulized to the esophagus^12^.

Second, in the case we reported, the CNS was also affected by the mycosis, which is rare: CNS involvement in PCM cases is only reported for 12.5% of the patients in large case series^17,18^. However, this type of involvement is being diagnosed more frequently in recent times, due to the advances of and improved accessibility to neuroimaging, provided the possibility of CNS involvement is kept in mind and actively sought^19^. CNS involvement may occur in up to 25% of PCM cases in CF^20^. Although the A/SAF is considered a more disseminated disease than the CF, CNS involvement appears to be more commonly associated with the latter, and in most cases previous lungs or upper respiratory tract involvement by the mycosis can be detected, signaling the mycotic nature of the SNC manifestations^17^. CNS-related manifestations vary depending on lesion type (parenchymatous or meningeal), extent and location^18,19^. The prompt diagnosis of CNS involvement is important for decisions on treatment, due to the inability of some antifungal drugs to penetrate the brain-blood barrier. In the case we reported, this was the rationale behind selecting a sulfa drug instead of itraconazole for the post-amphotericin B treatment. Although itraconazole should be the first-line choice according to the Brazilian guidelines, the selected sulfa drug penetrates the CNS more easily^1^. Noteworthy, our patient did not presented neurological symptoms or evidence of enhancing mass effects or perilesional edema, which suggests poor immunological reactivity. Potential reasons for this dampened immunoreactivity are discussed below.

The third point refers to the clinical form presented by our patient, a 56-year-old male smoker who resided in a rural area that is endemic for PCM in the Sao Paulo State for over 30 years. Due to his gardening hobby, he had a prolonged history of high-risk exposure to the fungus typical of the CF of PCM. However, surprisingly, an investigation of the extension of the disease, made during the patient’s hospitalization, revealed that he had not only typical symptoms of the CF of PCM, such as pulmonary involvement, but also features commonly found in the A/SAF, such as the enlargement of several superficial and deep lymphatic chains. Cases with concomitant clinical features of the CF and A/SAF of PCM had been described earlier, but commonly involved patients with a baseline immunosuppressive condition, such as Aids, cancer/chemotherapy or iatrogenic immunosuppression^21^.

The mechanisms driving PCM to the A/SAF or CF are still poorly known, mainly because of the difficulty in determining when the initial infection with *Paracoccidioides* sp*.* occurred in a given patient, and because the experimental models poorly mimic these events^22^. On the other hand, the immunological pattern that ultimately results from the development of either form has been better described in the literature. Distinct T cell reactivities are associated with the clinical outcomes of the disease. While the A/SAF is associated with overt Th2 immune responses and dampened Th-1 responses, the CF is associated with Th17 responses at the site of infection, with detectable but likely insufficient Th-1 responses, and, in its more severe cases, with a bias to Th-2 responses^23,24^.

The accumulation of information on patients with the mixed-form PCM shows that, although some clinical manifestations suggest an A/SAF of PCM, the illness is usually the result of reactivation of a chronic, subclinical infection instead. Currently, the accepted view is that the A/SAF of the disease results from the failure of the host’s immunity system in controlling the fungus spread from the early steps of the infection, which denotes a complete inability to mount effective Th-1 immune responses. On the other hand, in CF cases, there is partial control of fungus proliferation in the early phase of the disease, but later on (in years or even decades), the surviving fungi proliferate and spread, to cause the CF of the disease. In this case, there is certain degree of immune responsiveness, and, usually, a less uncontrolled disease compared with that of the A/SAF^1,2,23,24^. Therefore, the mechanism underlying the mixed form cases would be that these patients have a basal immunosuppressive condition which overrides the partial immunoreactivity and control of the disease displayed by the CF patients, allowing the fungus to proliferate and spread unrestrictedly as in the A/SAF of the disease. Therefore, we propose the hypothesis that a severe COVID-19 infection may have contributed to the development of an atypical and severe CF of PCM in our patient, similar to the mixed-form PCM described in immunosuppressed patients. In fact, besides experiencing well-recognized detrimental effects on innate immunity, which result in the abnormal release of inflammatory cytokines and other altered inflammatory responses, patients with severe COVID-19 experience T lymphopenia and a decrease in their number of NK cells^25,26^. Both of these cell types play crucial roles in the cell-mediated immune response to the fungus displayed by PCM patients^24,27^. Furthermore, patients with severe COVID-19 have an increased proportion of TCD4+ and TCD8+ cells exhibiting exhaustion phenotypes, which have been associated with impaired antiviral responses^28,29^: It is likely that the COVID-19 patients’ T cell-mediated antifungal responses will also be impaired. Evidencing this immune down regulation, COVID-19 has been associated with an increased risk of opportunistic fungal infections such as candidiasis, aspergillosis and, to a lesser extent, pneumocystis and mucormycosis^6^.

## CONCLUSION

We reported the case of a patient with severe SARS-CoV-2 infection and exacerbation of the clinical manifestations of an ongoing CF PCM, which resembled the mixed form of PCM described in immunosuppressed patients. This form of PCM may be related to the down regulation of the cell-mediated immune response caused by the severe SARS-CoV-2 infection. Therefore, different from the previous reports of COVID-19 in patients with PCM, our patient’s case suggests that this viral disease can influence the course of PCM in some patients. Our patient also presented other unusual manifestations of this mycosis, such as CNS and esophageal involvement. Fortunately, he was timely and appropriately treated for both diseases, evolving favorably.
